# Mitochondrial BK_Ca_ Mediates the Protective Effect of Low-Dose Ethanol Preconditioning on Oxygen-Glucose Deprivation and Reperfusion-Induced Neuronal Apoptosis

**DOI:** 10.3389/fphys.2021.719753

**Published:** 2021-10-25

**Authors:** Fang Su, Huajun Yang, Anchen Guo, Zhengyi Qu, Jianping Wu, Qun Wang

**Affiliations:** ^1^Department of Neurology, The Fourth Hospital of Harbin Medical University, Harbin, China; ^2^Department of Neurology, Beijing Tiantan Hospital, Capital Medical University, Beijing, China; ^3^China National Clinical Research Center for Neurological Diseases, Beijing, China; ^4^Collaborative Innovation Center for Brain Disorders, Beijing Institute of Brain Disorders, Capital Medical University, Beijing, China; ^5^Advanced Innovation Center for Human Brain Protection, Capital Medical University, Beijing, China

**Keywords:** mitoBK_Ca_ channel, ethanol preconditioning, oxygen-glucose deprivation and reperfusion (OGD/R), apoptosis, ischemia-reperfusion (I/R) injury, stroke

## Abstract

Ischemia-reperfusion (I/R) injury contributes to the morbidity and mortality of ischemic strokes. As an *in vitro* model, oxygen-glucose deprivation and reperfusion (OGD/R) exposure induces neuronal injury. Low-dose ethanol preconditioning (EtOH-PC) was reported to alleviate neuronal apoptosis during OGD/R. However, whether the mitochondrial BK_Ca_ (mitoBK_Ca_) channel is involved in the neuroprotective effect of EtOH-PC during OGD/R is not clearly defined. This study attempts to explore the mediation of the mitoBK_Ca_ channel in the neuroprotective effect of EtOH-PC on OGD/R-induced neuronal apoptosis and the underlying mechanisms. OGD/R model was established using primary cortical neurons that were preincubated with ethanol. Subsequently, the cell viability was measured by CCK-8 assay, and the apoptotic cells were determined by TUNEL assay. Annexin V/7-AAD staining and mitochondrial membrane potential using JC-10 were detected by flow cytometry. Western blot analysis was performed to check the apoptosis-related proteins. In the mixed primary culture, 95% neurofilament-positive cells were cortical neurons. Low-dose EtOH-PC (10 mmol/L) for 24 h significantly attenuated the OGD2h/R24h-induced neuronal apoptosis through activating the BK_Ca_ channel. Further investigations suggested that ethanol pretreatment increased the mitochondrial membrane potential (MMP) and downregulated the production of cleaved caspase 3 in OGD/R-injured neurons by activating the mitoBK_Ca_ channel. Low-dose ethanol pretreatment significantly attenuated the OGD/R-induced neuronal apoptosis mediated by the mitoBK_Ca_ channel which modulated the mitochondrial function by impeding the uncontrolled opening of mitochondrial permeability transition pore (MPTP).

## Introduction

Contributions of ischemia-reperfusion (I/R) to morbidity and mortality of ischemic strokes have been well documented (Yan et al., 2015). The oxygen-glucose deprivation and reperfusion (OGD/R) was used as a well-established *in vitro* model to induce neuronal apoptosis under pathological conditions and to study the neuroprotective effect of the pharmacological intervention (Alluri et al., 2015; Zhang et al., 2016). Apoptosis is defined as a form of programmed cell death maintaining the stabilization of the intracellular environment under physiological or pathological conditions, and the apoptotic pathway is divided into two types: the extrinsic pathway and the intrinsic pathway. The extrinsic pathway mainly relies on the binding of death ligands to their receptor localized on the cell membranes. On the contrary, the intrinsic pathway was mediated by the mitochondrial depolarization and the subsequent release of cytochrome C and other large molecules, leading to the activation of caspases and the formation of apoptotic bodies (Burg et al., 2006). Therefore, pharmacological intervention, such as metformin or neuroserpin, might protect neurons from OGD/R-induced damage (Meng et al., 2016; Yang et al., 2016).

The large-conductance, Ca^2+^-activated K^+^ channels (BK_Ca_) is one of the intrinsic molecular determinants that regulate neuronal excitability and neurotransmitter release in the central nervous system (CNS) (Raffaelli et al., 2004). The protective effect of BK_Ca_ channels from I/R injury has been well documented by both transgenic animal models and pharmacological interventions. For instance, resveratrol treatment reversed neuronal damage induced by OGD by activating BK_Ca_ channels (Zhang et al., 2008). Furthermore, universal expression and localization of BK_Ca_ channels on the membranes of both plasma and mitochondria were observed (Bednarczyk et al., 2013). In addition, a recent report has demonstrated mitochondrial BK_Ca_ (mitoBK_Ca_) as a member of the *Kcnma1* gene family (Singh et al., 2013), roles of which have been well characterized in protecting the heart from ischemia, properly through regulation of the generation of reactive oxygen species (ROS), mitochondrial Ca^2+^ flux, and the permeability of the mitochondrial membrane (Xu et al., 2002; Balderas et al., 2015). However, whether mitoBK_*C*__*a*_ is involved in the neuronal apoptosis induced by OGD/R is not clearly understood.

Ethanol consumption, specifically low-to-moderate ethanol intake, may typically initiate the cytoprotective mechanism that prevents the deleterious effects of subsequent I/R (Yamaguchi et al., 2002; Korthuis, 2004) as an effect of ethanol preconditioning (EtOH-PC) and finally may reduce the risk of stroke morbidity and mortality (Reynolds et al., 2003; Ducroquet et al., 2013; Zhang et al., 2014). The cardioprotective and neuroprotective effects of ethanol against I/R injury have been proposed by the famous “French paradox” (Sun et al., 2002), which was consistent with our previous work to explore the neuroprotective effect of ethanol in a gerbil model (Wang et al., 2007). Moreover, another study from our group demonstrated the potential correlation between ethanol and the activation of BK_Ca_ channel in an *in vitro* OGD/R model (Su et al., 2017). However, the mitoBK_Ca_-involved neuroprotective effect of EtOH-PC is not clearly defined.

Here, we showed that low-dose EtOH-PC significantly attenuated the OGD/R-induced neuronal apoptosis mediated by mitoBK_Ca_ channel which modulated the mitochondrial function by impeding the uncontrolled opening of MPTP. This study identified mitoBK_Ca_ as a promising target for the neuroprotective treatment of ischemic stroke. More importantly, the low-lose EtOH-PC is beneficial for stroke patients and is valuable for further study in clinics as the mitoBK_Ca_ activator.

## Materials and Methods

### Primary Cortical Neurons Culture

Isolation of the primary neurons from Sprague-Dawley rat fetuses (Vital River Laboratory Animal Technology Co., Ltd., Beijing, China) has been described previously (Liu Y. et al., 2012) with minor modification. All the experiments have been approved by the Ethics Committee of the Beijing Tiantan Hospital of Capital Medical University. The bilaterally cortical brain tissue was collected and minced, followed by digestion with 0.125% trypsin EDTA supplemented with 0.5 mg/ml DNase for 15 min at 37°C, and were then terminated by mix with complete medium containing DMEM/F12 with 10% fetal bovine serum and 5% horse serum. Single-cell suspension was then isolated by centrifugation followed by resuspension with culture medium (DMEM/F12, 10% FBS, 5% HS, 0.5 mmol/L L-glutamine, and 1% penicillin/streptomycin) into the plates coated with poly-L-lysine (0.1 g/L). After 4 h incubation, the medium was changed with a medium composed of neurobasal-R medium, 2% B27, 1% BSA, and 1% penicillin/streptomycin. The cells were maintained at 37°C in an incubator supplemented with 5% CO_2_.

### Immunofluorescence

Immunofluorescence was performed to confirm the purity of the isolated neuron cells as previously described (Su et al., 2017). Briefly, cells were first fixed with 4% paraformaldehyde after culturing for 7 days, and then, they were stained with anti-neurofilament antibody and anti-glial fibrillary acidic protein antibody to label the endogenous expression of both proteins at 4°C overnight (1:500, Beijing GuanXing Yun Science and Technology Co., Ltd., Beijing, China). The next day, the unbind primary antibodies were removed by washing, and then, the cells were incubated with secondary antibodies at room temperature for about 1 h. The unbind secondary antibodies were removed as previously described, and then, the DNAs were stained with DAPI for 2 min at room temperature. Cell images were collected using a microscope from Olympus.

### Oxygen-Glucose Deprivation and Reoxygenation

To establish the OGD/R mode, neuron cells were first challenged with glucose-free DMEM (Thermo Fisher Scientific, Inc., Waltham, MA, United States) and a hypoxic condition with 5% carbon dioxide, 2% oxygen, and 93% nitrogen at 37°C for 1, 2, and 3 h, respectively, and then, they were recovered with culture medium with normal glucose as well as normoxic condition with 5% carbon dioxide at 37°C for 24 h (reoxygenation period).

### Pharmacological Treatments

Cells were first treated with paxilline at 5 μmol/L for 10 min, and then, they were incubated with 10 mmol/L ethanol (Sigma-Aldrich, St. Louis, MO, United States) for 24 h followed by OGD/R.

### Cell Viability Assay

The cell viability was measured by Cell Counting Kit-8 (CCK-8; Dojindo, Kumamoto, Japan) Briefly, 20 μl of the CCK-8 reagent was mixed with the culture medium and then incubated at 37°C for about 4 h. The absorbance at OD 450 was measured by the Molecular Device M5.

### Electrophysiology Recording

Borosilicate glass patch pipettes for single-channel recordings had a resistance of 3–5 MX when filled with an internal solution. Recordings were made using a patch clamp amplifier and patch master 2.73 amplifier (Heka, Lambrecht, Pfalz, Germany). The single-channel recordings were filtered at 1–5 kHz and digitized at 20 kHz. All experiments were performed at room temperature (22–25°C). Fitmaster software (Heka, Lambrecht, Pfalz, Germany) was used for data analysis. Open probability is expressed as channel open probability (NPo), where N represents the number of single channels present in the patch, and Po is the open probability of a single channel. NPo was calculated as follows: NPo = (A_1_ + 2 A_2_ + 3 A_3_ + n An)/(A_0_ + A_1_ + A_2_ + …An), where A_0_ is the area below, the plot of the amplitude histogram corresponds to the closed state, and A_1_–An represents the area of the histogram, reflecting different open current levels from 1 to n channels in the patch.

### Terminal Deoxynucleotidyl Transferase (TdT)-Mediated dUTP Nick End-Labeling (TUNEL)

TUNEL assay for the detection of apoptosis was performed according to the instruction of the manufacturer (Thermo Fisher Scientific, Waltham, MA, United States). Apoptotic cells were pictured by microscope (Olympus), and counted and analyzed by Graphpad Prism 6.0 from five independent fields.

### Annexin V-PE and 7-AAD Double-Staining Assay

Apoptosis was determined using the PE Annexin V Apoptosis Detection Kit with 7-AAD (BioLegend) and detected by flow cytometry (BD C6; BD Biosciences, Franklin Lakes, NJ). Briefly, the cells were collected by trypsinization and then washed with PBS three times to remove the debris and complete medium. Subsequently, cells were stained with 5 μl of Annexin V-PE and 5 μl of 7-AAD at dark for 15 min at 23–25°C. For the analysis of the apoptotic cells at different stages as well as the non-apoptotic cells, the early apoptotic cells were defined as positive for PE-Annexin-V and negative for 7-AAD, whereas the late-stage apoptotic cells were defined as positive for PE-Annexin V as well as 7-AAD.

### Detection of Mitochondrial Membrane Potential

After treatment, the neuronal cells were collected by trypsinization and then labeled with JC-10 (Beyotime Biotechnology, Shanghai, China) at 37°C for 30 min. Next, the cells were centrifuged at 1,000 rpm for 4 min, followed by washing with PBS three times. The cells were then suspended with 400 μl flow buffer and were analyzed by flow cytometry (BD C6; BD Biosciences, Franklin Lakes, NJ).

### Western Blot

Total cell lysate was prepared from neuronal cells with radioimmunoprecipitation assay buffer (RIPA) supplemented with protease inhibitor (Biochem, PA, United States). Cell concentrations were quantitated by BCA assay and were denatured with SDS-based sample buffer. In Western blot, equal amounts (30 μg) of protein were loaded and separated by SDS-PAGE and then transferred onto the PVDF membrane. The blot was first blocked with 5% non-fat milk and then incubated with primary antibody overnight at 4°C. Antibodies for cleaved caspase 3 (1:1,000; Cell Signaling Technology, MA, United States) and total caspase 3 (1:1,000; Cell Signaling Technology, MA, United States), anti-Drp1 (ab184247), and anti-Fis1 (ab156865) were purchased from Abcam, and anti-March 5 (19168) was purchased from Cell Signaling Technology. Next, the blots were washed with TBST and then incubated with secondary antibodies conjugated with HRP at room temperature for 1 h. Signals were collected by adding ECL solution and were captured by the FluorChem FC2 System (Cell Biosciences, Inc., Santa Clara, CA, United States), and images were analyzed using ImageJ software (NIH, United States).

### Mitochondrial Permeability Transition Pore Assay

All the cells were first collected by trypsinization and were washed with PBS two times, and then, they were resuspended with buffer containing Calcein AM, quenching solution. The concentration of the cells was 1×10^6^ ml^–1^, and the cells were kept at 37°C for 30 min. The cells were centrifuged at 1,000 *g* for 5 min and then resuspended with detection buffer for flow cytometry analysis.

### Statistical Analysis

All the data were expressed as mean ± standard deviation (S.D.) and were analyzed with SPSS version 25.0. Significant differences were determined by ANOVA followed by Dunnett’s multiple comparison test, and the statistical significance was defined as *P* < 0.05.

## Results

### Identification of Primary Cortical Neurons

**FIGURE 1:**
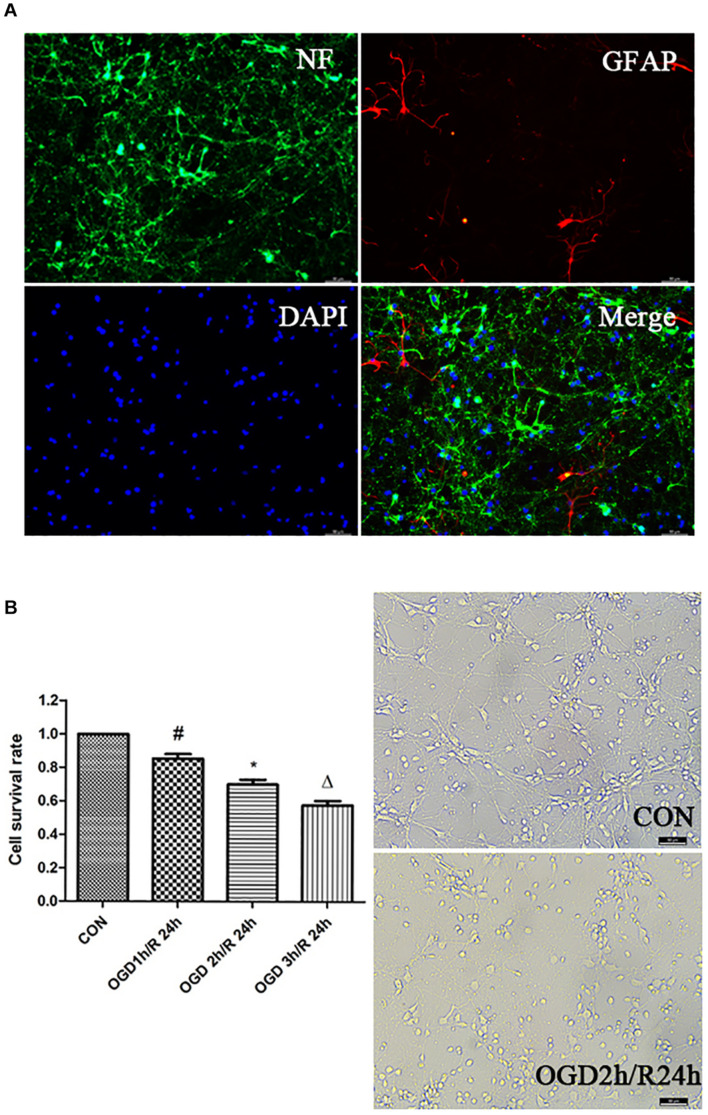
Establishment of OGD/R model. **(A)** Identification of primary cortical neurons. Double-labeling immunofluorescence staining with the neuron marker NF (green) and the astrocyte marker GFAP (red); nuclei stained with DAPI; scale bar: 50 μm. **(B)** Effects of different time of OGD/R on neuron viability. Cell viability was measured by CCK-8 assay. ^#^*P* < 0.01 vs. control; *P < 0.01 vs. control; ^Δ^*P* < 0.01 vs. control.

To examine the purity of cortical neurons in the primary mixed culture, we stained the cells with anti-NF directed against the neurofilament protein and anti-GFAP directed against glia-specific glial fibrillary acidic protein and observed that 95% NF-positive cells were cortical neurons (Figure 1A). To determine the optimal OGD/R condition, we exposed the neurons to oxygen-glucose deprivation for 1, 2, or 3 h, respectively, and followed by reperfusion for 24 h. The neurons viability was decreased with an increase in the deprivation time, and we determined 2 h OGD as the optimal deprivation time (Figure 1B).

### BK_Ca_ Mediated the Protective Effect of Ethanol Preconditioning Against Oxygen-Glucose Deprivation and Reperfusion Injury

**FIGURE 2:**
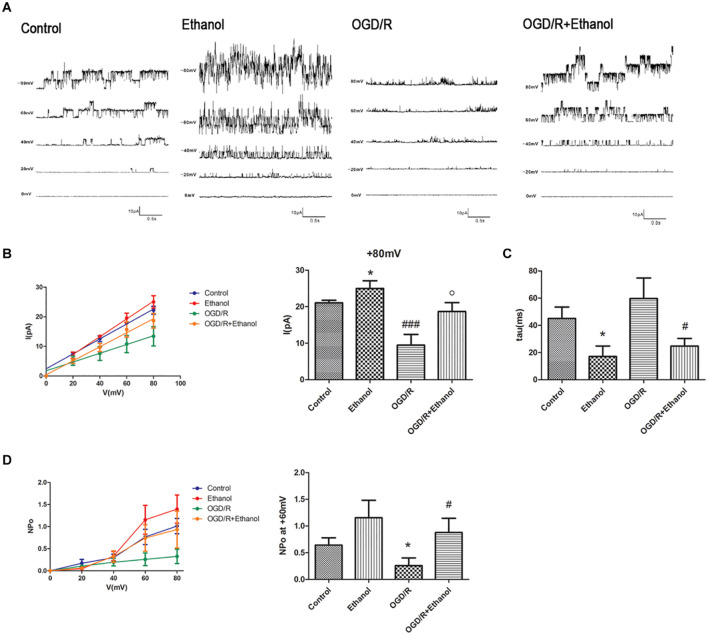
BK_Ca_ mediated the protective effect of ethanol preconditioning. **(A)** Representative single-channel recordings of BK_Ca_ channels in the inside-out configuration. **(B)** The current–voltage curve of the BK_Ca_ channels. *P < 0.05 vs. control; ^###^*P* < 0.001 vs. control; ^*o*^*P* < 0.05 vs. OGD/R. **(C)** Statistical data for close time of the BK_Ca_ channels. *P < 0.05 vs. control; ^#^*P* < 0.05 vs. OGD/R. **(D)** Statistical data for open probability (NPo) of the BK_Ca_ channels. *P < 0.05 vs. control; ^#^*P* < 0.05 vs. OGD/R.

In view of our previous findings that 10 mmol/L ethanol treatment could activate BK_Ca_ channels at all membrane voltages (Su et al., 2017), we then investigated whether EtOH-PC reversed the OGD/R-induced neuronal injury through activating BK_Ca_ channel. We examined the current–voltage and conductance of BK_Ca_ channel using an inside-out patch after 10 mmol/L EtOH-PC followed by OGD2h/R24h. It suggested that ethanol increased the current and conductance of BK_Ca_ and decreased the close time of BK_*Ca.*_ OGD/R exposure markedly decreased the current and conductance of BK_Ca_ and increased the close time of BK_Ca_. The NPo of BK_Ca_ was decreased under OGD/R, while low-dose EtOH-PC counteracted the effect of OGD/R on current, conductance, close time, and NPo of BK_Ca_ (Figure 2).

### Ethanol Preconditioning Protected Against the Oxygen-Glucose Deprivation and Reperfusion-Induced Neuronal Apoptosis Through Activating BK_Ca_ Channel

**FIGURE 3:**
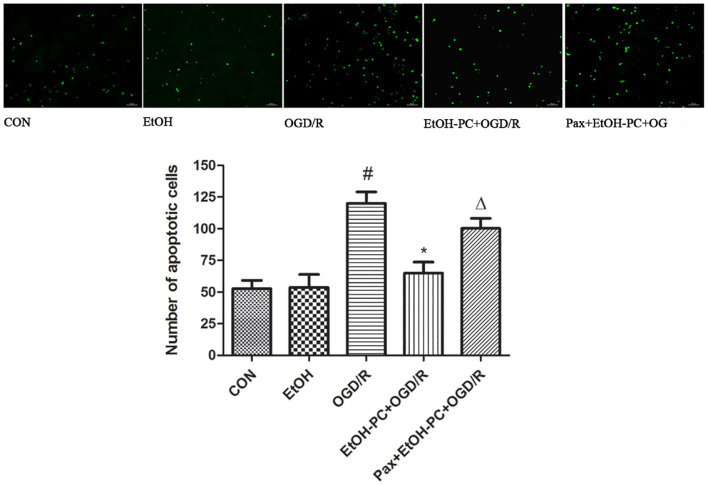
Ethanol preconditioning protected against the OGD/R-induced neuronal apoptosis through activating BK_Ca_ channel detected by TUNEL staining. Results are expressed as mean ± S.D. (*n* = 5). ^#^*P* < 0.01 vs. CON; *P < 0.01 vs. OGD/R; ^Δ^*P* < 0.05 vs. EtOH-PC + OGD/R; scale bar: 100 μm.

Since OGD/R decreased the neuronal viability and ethanol dramatically attenuated the OGD/R-induced neuronal injury, we next investigated whether apoptosis was involved in the decreased cell viability and whether ethanol could attenuate the OGD/R-induced neuronal apoptosis. As expected, OGD/R exposure led to apoptosis in neurons as demonstrated by the TUNEL assay. Furthermore, EtOH-PC had no effect on the normoxic neurons, but markedly reduced the apoptotic cells after OGD/R exposure. BK_Ca_ channel blocker paxilline preincubation counteracted the protective effect of ethanol (*P* < 0.01, Figure 3).

**FIGURE 4:**
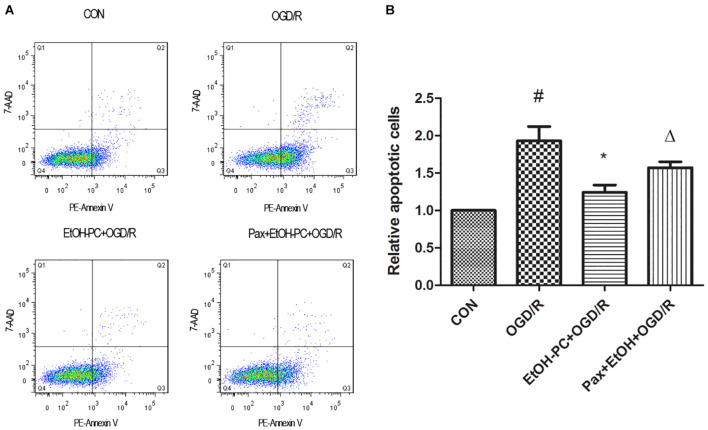
Ethanol preconditioning protected against the OGD/R-induced neuronal apoptosis through activating BK_Ca_ channel detected by flow cytometry. After the neurons were preincubated with paxilline (5 μmol/L) for 10 min and preconditioned with ethanol (10 mmol/L) followed by OGD2h/R24h, the neurons were stained with Annexin V and 7-AAD and detected by flow cytometry **(A)**. Results are expressed as mean ± S.D. (*n* = 5). ^#^*P* < 0.01 vs. CON; *P < 0.05 vs. OGD/R; ^Δ^*P* < 0.05 vs. EtOH-PC + OGD/R **(B)**.

In addition, the neuron apoptosis was further confirmed by flow cytometry after staining with Annexin V-7-AAD, indicating that EtOH-PC dramatically reduced the apoptotic cells. Compared with EtOH + OGD/R group, BK_Ca_ channel blocker paxilline preincubation significantly counteracted the neuroprotective effect of EtOH-PC (Figures 4A,B).

Taken together, low-dose EtOH-PC protected against OGD/R-induced neuronal apoptosis through activating BK_Ca_ channel.

### Ethanol Preconditioning Decreased Mitochondrial Membrane Potential Through Activating Mitochondrial BK_Ca_

**FIGURE 5:**
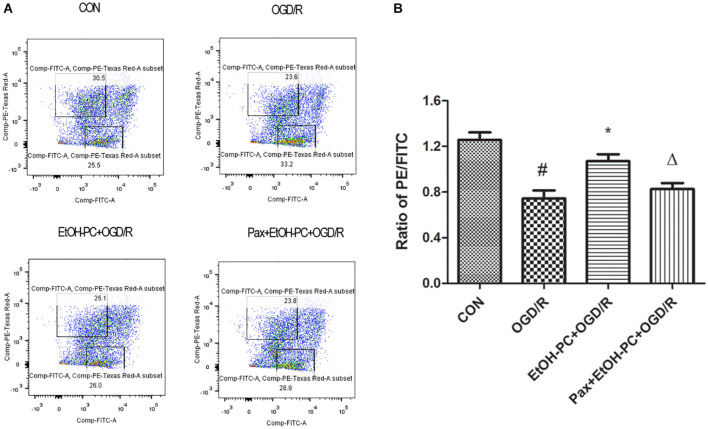
Ethanol preconditioning increased the mitochondrial membrane potential through activating mitoBK_*Ca.*_ After the neurons were preincubated with paxilline (5 μmol/L) for 10 min and preconditioned with ethanol (10 mmol/L) followed by OGD2h/R24h, the neurons were incubated with JC-10 at 37°C for 30 min. Then, the cells were centrifuged, and the stained cells were subjected to flow cytometry assay **(A)**. Results are expressed as mean ± S.D. (*n* = 5).^ #^*P* < 0.01 vs. CON; *P < 0.05 vs. OGD/R; ^Δ^*P* < 0.05 vs. EtOH-PC + OGD/R **(B)**.

**FIGURE 6:**
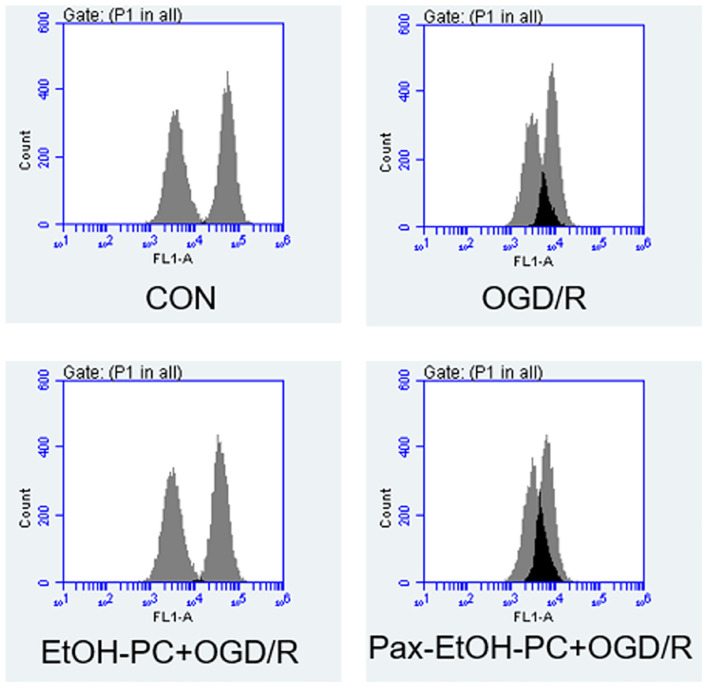
Ethanol preconditioning regulated the mitochondrial permeability transition pore during OGD/R. The neuron cells were preincubated with paxilline (5 μmol/L) for 10 min and preconditioned with ethanol (10 mmol/L) followed by OGD2h/R24h. The mitochondrial potential was determined by mitochondrial permeability transition pore (MPTP) assay.

To determine whether the mitochondrial BK_Ca_ channel was involved in the protective effect of EtOH-PC, we examined the mitochondrial membrane potential using flow cytometry. Compared with the control group, OGD/R exposure partially opened MPTP and subsequently decreased the mitochondrial membrane potential. EtOH-PC closed MPTP and increased the MMP. However, BK_Ca_ channel blocker paxilline preincubation before ethanol significantly counteracted the effect of ethanol with the decrease of MMP (Figures 5A,B). In addition, a similar trend of regulation was further confirmed by the MPTP assay (Figure 6). It suggested that mitoBK_Ca_ mediated the protective effect of EtOH-PC.

### Ethanol Preconditioning Downregulated Intrinsic Apoptosis-Related Proteins Through Activating Mitochondrial BK_Ca_

**FIGURE 7:**
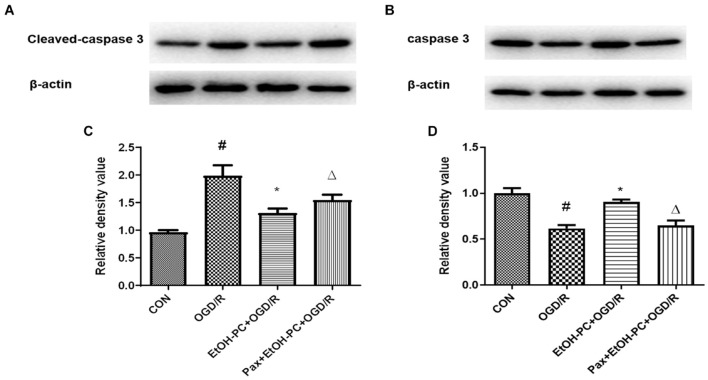
Ethanol preconditioning downregulated the cleaved caspase 3 expression through activating mitoBK_Ca_. After the neurons were preincubated with paxilline (5 μmol/L) for 10 min and preconditioned with ethanol (10 mmol/L) followed by OGD2h/R24h, cleaved caspase 3 and total caspase 3 were detected by Western blot analysis. Representative blots in **(A)** cleaved caspase 3 and **(B)** total caspase 3. Quantification of the ratio of cleaved caspase 3 to β-actin **(C)** and total caspase 3 to β-actin **(D)**. Results are expressed as mean ± S.D. (*n* = 3). ^#^*P* < 0.01 vs. CON; *P < 0.01 vs. OGD/R; ^Δ^*P* < 0.01 vs. EtOH-PC + OGD/R **(C)**. ^#^*P* < 0.01 vs. CON; *P < 0.01 vs. OGD/R; ^Δ^*P* < 0.01 vs. EtOH-PC + OGD/R **(D)**.

**FIGURE 8:**
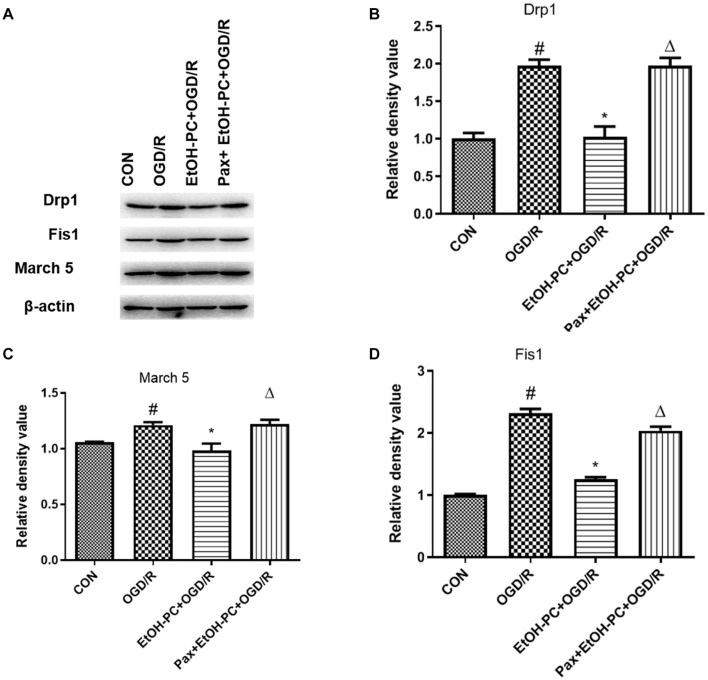
Ethanol preconditioning downregulated intrinsic apoptosis markers during OGD/R through activating mitoBK_Ca_. After the neurons were preincubated with paxilline (5 μmol/L) for 10 min and preconditioned with ethanol (10 mmol/L) followed by OGD2h/R24h. **(A)** Representative blots of Drp1, Fis1, and March 5, and the quantification result were shown in **(B–D)**. Results are expressed as mean ± S.D. (*n* = 3). ^#^*P* < 0.001 vs. CON; *P < 0.01 vs. OGD/R; ^Δ^*P* < 0.01 vs. EtOH-PC + OGD/R **(B)**. ^#^*P* < 0.01 vs. CON; *P < 0.05 vs. OGD/R; ^Δ^*P* < 0.05 vs. EtOH-PC + OGD/R **(C)**. ^#^*P* < 0.001 vs. CON; *P < 0.001 vs. OGD/R; ^Δ^*P* < 0.001 vs. EtOH-PC + OGD/R **(D)**.

When the mitochondrial membrane potential decreases, caspase 3 is cleaved and activated by the apoptosis-inducing factor released from the mitochondrial extracellular compartment and apoptotic protease activating factor-1 released from mitochondria. So, we examined the cleaved caspase 3 expression and found that OGD/R exposure upregulated the cleaved caspase 3 expression. EtOH-PC downregulated the cleaved caspase 3 expression. BK_Ca_ channel blocker paxilline significantly counteracted the downregulation effect of ethanol (Figure 7). In addition, markers (Drp1, Fis1, March 5) for the apoptosis pathway were analyzed by Western blot, and the results suggested that ethanol-mediated neuroprotective effect was dependent on the intrinsic apoptosis pathway, as can be seen from Figure 8. Collectively, our data demonstrated that mitoBK_Ca_ channel activation mediated the protective effect of EtOH-PC.

## Discussion

In this study, our findings suggested that OGD/R-induced neuronal apoptosis was mediated by BK_Ca_ channel. Low-dose EtOH-PC significantly attenuated the OGD/R-induced neuronal apoptosis through activating BK_Ca_ channel. Further mechanism investigations suggested that mitochondrial BK_Ca_ (mitoBK_Ca_) mediated the neuroprotective effect of EtOH-PC by impeding the uncontrolled opening of MPTP and modulating the mitochondrial function.

The BK_Ca_ belongs to the K_Ca_ family and is activated by multiple signals including elevated levels of intracellular Ca^2+^ and membrane depolarization, leading to a large K^+^ conductance. As a consequence, the membranes were re/hyperpolarized, and the voltage-dependent Ca^2+^ channels were closed (Bednarczyk et al., 2013). BK_Ca_ channel is involved in the hyperglycemia-altered apoptosis and proliferation in HEK293 cells (Chang et al., 2011). Activation of BK_Ca_ channels elicits the infarct-sparing effects of late ischemic preconditioning in myocardial I/R injury in animal models (Wang et al., 2010). Liu X.R. et al. (2012) suggested that propofol causes greater vasodilating effects by increasing the Ca^2+^ sensitivity of BK_Ca_ channel in the cerebral arterial smooth muscle cells. In this study, the single-channel recordings of the inside-out patch clamp showed that the BK_Ca_ channel was deactivated by OGD/R in cortex neuron cells; more importantly, low-dose (10 mmol/L) ethanol activated BK_Ca_ channels suggested that BK_Ca_ is a promising target for I/R injury treatment, and low-dose ethanol might be the BK_Ca_ channel opener.

Mitochondria are key organelles defining cell fate. The inner mitochondrial membrane is particularly vital because it contains the respiratory chain complex, which makes the mitochondria not only an ATP producer but also a regulator of redox homeostasis and Ca^2+^ (Balderas et al., 2015). More and more K^+^ selective channels have been uncovered in the internal membrane, such as mitoBK_Ca_. mitoBKCa may regulate mitochondrial function as a redox sensor (Augustynek et al., 2014). The opening of the mitoBK_Ca_ channel to a certain extent can protect the mitochondria from the uncontrolled MPTP opening, leading to the increase of mitochondrial membrane potential and the inhibition of apoptosis (Cheng et al., 2008, 2011). The mitochondrial cation channel BK_Ca_ plays a significant role in cardioprotection from I/R injury (Ponnalagu and Singh, 2016). However, it is not clearly understood whether mitoBK_Ca_ is involved in the neuroprotection of ethanol from OGD/R-induced apoptosis in I/R injury.

Recently, brain preconditioning, especially pharmacological preconditioning, has demonstrated promising benefits as a novel treatment option for ischemic stroke (Stetler et al., 2014). Although excessive ethanol drinking is highly correlated with increased stroke risk (Balderas et al., 2015) and severity (Ducroquet et al., 2013), multiple lines of evidence suggest that low-to-moderate ethanol consumption (1–2 beverages or 30 g of ethanol per day) may exert a protective effect against I/R injury (Yamaguchi et al., 2002; Korthuis, 2004). Our previous study demonstrated that low-dose ethanol protected against OGD/R-induced neuronal injury. As described in this study, we observed the OGD/R-induced neuronal apoptosis determined by Annexin V and 7-AAD staining, as well as TUNEL staining. Low-dose EtOH-PC significantly attenuated the OGD/R-induced neuronal apoptosis through activating the BK_Ca_ channel.

In mitochondria, when the mitochondrial membrane potential decreases, caspase 3 is cleaved and activated by the apoptosis-inducing factor released from the mitochondrial extracellular compartment and apoptotic protease activating factor-1 released from mitochondria. Once activated, procaspase-3 is cut into cleaved caspase-3 and plays the role of proteolytic enzymes promoting apoptosis (Lazebnik et al., 1994). In this study, we demonstrated that OGD/R induced the decrease of mitochondrial membrane potential and upregulated the cleaved caspase 3 expression. Low-dose EtOH-PC increased the mitochondrial membrane potential and downregulated the cleaved caspase 3 expression, leading to the inhibition of apoptosis. After inhibiting the BK_Ca_ channel by paxilline, the protective effects of ethanol were counteracted, suggesting that the mitoBK_Ca_ channel mediated the protective effect of low-dose EtOH-PC against the OGD/R-induced neuronal apoptosis.

Taken together, our study has identified mitoBK_Ca_ as a critical channel that mediates the protective effect of low-dose EtOH-PC against the neuronal apoptosis induced by OGD/R. mitoBK_Ca_ might be a promising target for the neuroprotective treatment of ischemic stroke. As a potential mitoBK_Ca_ activator, the protective effect of low-dose EtOH-PC in human stroke patients warrants future investigation.

## Data Availability Statement

The raw data supporting the conclusions of this article will be made available by the authors, without undue reservation.

## Ethics Statement

The animal study was reviewed and approved by the Animal Care and Use Committee of Capital Medical University.

## Author Contributions

All authors listed have made a substantial, direct and intellectual contribution to the work, and approved it for publication.

## Conflict of Interest

The authors declare that the research was conducted in the absence of any commercial or financial relationships that could be construed as a potential conflict of interest.

## Publisher’s Note

All claims expressed in this article are solely those of the authors and do not necessarily represent those of their affiliated organizations, or those of the publisher, the editors and the reviewers. Any product that may be evaluated in this article, or claim that may be made by its manufacturer, is not guaranteed or endorsed by the publisher.
